# Ultra Large Gene Families: A Matter of Adaptation or Genomic Parasites?

**DOI:** 10.3390/life6030032

**Published:** 2016-08-08

**Authors:** Philipp H. Schiffer, Jan Gravemeyer, Martina Rauscher, Thomas Wiehe

**Affiliations:** 1 Institute for Genetics, University of Cologne, Köln 50674, Germany; jgraveme@smail.uni-koeln.de (J.G.); martina.rauscher@uni-koeln.de (M.R.); 2 Division of Biosciences, GEE, University College London, London WC1E 6BT, UK

**Keywords:** gene family, genome evolution, adaptation, neutral evolution, selection, NLR-genes, run-away-evolution, gene clusters

## Abstract

Gene duplication is an important mechanism of molecular evolution. It offers a fast track to modification, diversification, redundancy or rescue of gene function. However, duplication may also be neutral or (slightly) deleterious, and often ends in pseudo-geneisation. Here, we investigate the phylogenetic distribution of ultra large gene families on long and short evolutionary time scales. In particular, we focus on a family of NACHT-domain and leucine-rich-repeat-containing (NLR)-genes, which we previously found in large numbers to occupy one chromosome arm of the zebrafish genome. We were interested to see whether such a tight clustering is characteristic for ultra large gene families. Our data reconfirm that most gene family inflations are lineage-specific, but we can only identify very few gene clusters. Based on our observations we hypothesise that, beyond a certain size threshold, ultra large gene families continue to proliferate in a mechanism we term “run-away evolution”. This process might ultimately lead to the failure of genomic integrity and drive species to extinction.

## 1. Rationale

Gene duplication is recognised since many decades as a key mechanism of evolution [1,2,3,4]. It has been found to be relevant for the major evolutionary transitions, for speciation, for opening new physiological and ecological opportunities and for short time-scale adaptation. Gene duplication can alter gene dosage, rescue gene function, and lead to evolution of new genetic networks, and to the re-wiring or modulation of existing ones. While it is still unclear if the first cellular organisms had many or few genes, it seems plausible to assume that all genes were initially present in a single copy, and it is clear that present-day gene families are a result of ongoing, and lineage-specific, duplication and loss. Whether this process is mostly neutral, or driven by natural selection, is controversial [4,5,6]. It can act on short evolutionary time-scales and gene family sizes can substantially differ even between closely related sister species, as for instance observed in *Drosophila* [7]. Many more examples are found in the vast literature on gene duplication and multi gene families, see for example [8]. Here, we focus on very large families and pay special attention to tandemly arrayed gene clusters in these families. 

One massive expansion of such a family, supposedly acting in innate immunity, was recently described in the zebrafish [9]. Based on an initial analysis by Stein et al. [10], about 400 NLR-B30.2 genes have been identified in the fish genome. Only a small part of them are found as orthologues in the closely related carp genome. Curiously, the vast majority (227) of the zebrafish NACHT-domain and leucine-rich-repeat-containing (NLR) genes are densely packed on one arm of chromosome 4, interspersed with a family of diverse, but so far not characterised, zinc finger genes. Such clusters are a consequence of tandem duplication and non-homologous cross-over. Different rates and patterns of subsequent gene conversion can lead to both diversification or homogenisation, in particular of young paralogues [9,11]. The existence of large clusters of duplicated genes is known not only from vertebrates, but also from other eukaryotes, for example the nematode model organism *Ceanorhabditis elegans* [12]. Many of the clustered gene families found in this nematode appeared to be lineage specific expansions (“in-paralogues”) and the ones in the largest clusters were tentatively connected to environmental interaction, for instance the P450, and F-Box genes, or the G-coupled receptors [12].

However, to our knowledge, such an ultra large multi gene family as the NLR-B30.2 genes in zebrafish, with one large cluster dominating a whole chromosomal arm, has not been described before in Metazoa. Thus, it is an obvious question to ask whether this is a singular case in zebrafish or if there are other examples of huge gene-families, with hundreds of members, which are organised in genomic clusters.

The sheer number of genes found in some of these clusters also leads to the question if functional diversification, potentially in some domains, is beneficial and the inflation of the family is selectively favoured. Alternatively, most of the duplicates could be redundant and neutral, or even deleterious, for the organism. Thus, these genes should be prone for pseudo-geneisation and eventual loss. Another possibility is that the ultimate selfish gene, like transposable elements, is one that is propagating itself not only vertically, across time, but also laterally, across the genome, as a genomic parasite.

The latter is again especially interesting in the zebrafish as most of the NLR genes not residing on chromosome 4 are clustered within 15% of the ends of other chromosomes. A prevalence for insertion in telomeric regions is known for transposable elements [13]. It is thus conceivable that NLR genes from chromosome 4 are spawned into other regions of the zebrafish genome, where they proliferate into new clusters.

## 2. Results

To form an opinion about how special the situation in the zebrafish genome truly is, we conducted a survey in other species with well annotated genomes to search for families with large sets of clustered genes. NLR genes are in particular interesting for this, as they have also been reported as expanded in phylogenetically very different groups of animals, such as the sea urchin *Strongilocentrotus purpuratus* and the sponge *Amphimedon queensladica* [14,15]. While one can analyse the independent evolution of these genes in different organisms, it is unfortunately not possible to trace this gene family through well-annotated model species genomes. They were neither described in large numbers in the model genera *Caenorhabditis*, nor in *Drosophila*, nor the great apes. To get an overview of ultra-large gene families we decided to not exclusively concentrate on NLR family genes but to also include other large gene families. 

We analysed a set of phylogenetically distant species: the acraniate *Branchiostoma floridae*, the coral *Acropora digitifera*, the sponge *Amphimedon queenslandica*, the carp *Cyprinus carpio*, and the sea urchin *Strongylocentrotus purpuratus*. We also re-analysed several fish species (see [9] for initial analysis), which are separated by a few hundred Myr of divergence, namely the pike *Esox lucius*, the torafugu *Takifugu rubripes*, the cichlid *Maylandia zebra*, the spotted gar *Lepisosteus oculatus*, the cavefish *Astianax mexicanus*, and the model organisms medaka *Oryzias latipes* and killifish *Nothobranchius furzeri*. We complemented this with comparisons on shorter time scales by analysing selected taxa of animals from primates, nematodes and diptera, containing the model organisms *Caenorhabditis elegans*, *Drosophila melanogaster* and *Homo sapiens.* Details are described in Methods. 

In *C. elegans* inflations of F-box and T-box transcription factors, and of nuclear hormone receptors have been reported [16]. We were interested to see if these genes expanded throughout the genus or only in lineages leading to single species. To complement manual data screening of the InterProScan output, we applied a Fisher’s exact test to test for enrichment of Pfam domains in pairwise species comparisons. We performed pairwise comparisons of 15 species to identify additional cases of species-specific gene expansions (Table 1). Using this method, we recovered, for example, F-box genes as extremely expanded in *C. remanei.* Another example are fibronectins in human (*n* = 970). However, these 970 human fibronectin domains collapsed into 123 genes widely dispersed throughout the entire genome when we removed redundancy. 

**Figure 1:**
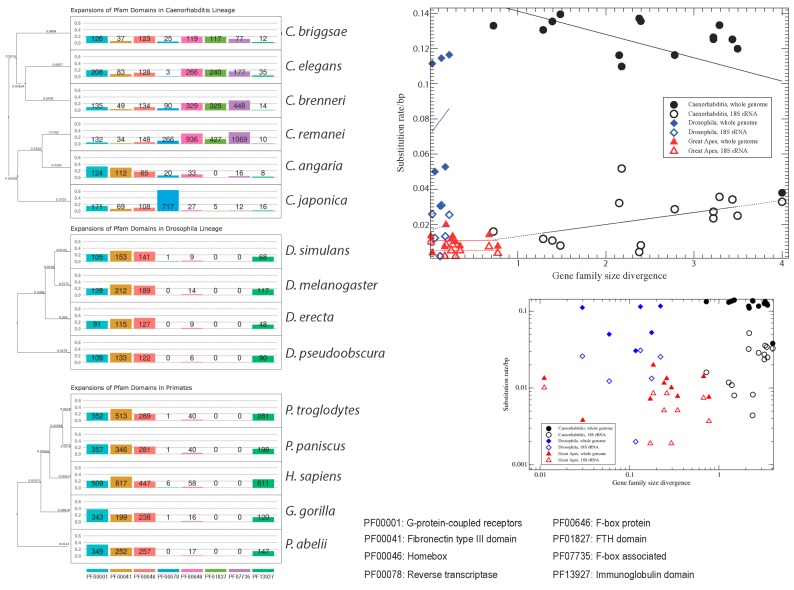
Domains and proteins with large families in *Caenorhabditis*, *Drosophila*, and the great apes. Trees are based on NCBI taxonomy with branch lengths scaled for divergence time following [14,15,16]; grey branches without divergence time estimate. Gene family size divergence calculated for any pair of species as mean squared difference in gene counts. Per nucleotide pairwise substitution rates are calculated with the program Andi [17]. Axes scaling is linear (large plot) and logarithmic (small plot). Linear regression lines are shown only in the large plot.

Manually mining the InterProScan output, we divided the inflated gene families into three different size fractions: 150–200 genes, 200–400 genes, and more than 400 genes (Figure 1). Comparing the divergence in gene family sizes (see Methods) to substitution rates on branches leading to single species in all three analysed taxa (genera *Caenorhabditis* and *Drosophila*, and the great apes; Table 2) we found no correlation between these two measures. In *Drosophil*a we observe Pearson’s correlation between family size divergence and substitution rates of *r* = 0.12 (0.82) for the whole-genome comparison and of *r* = 0.08 (0.87) (*p*-values in parentheses) for the single-gene (SSU 18S rRNA) comparison. In the great apes we measured *r* = −0.35 (0.33) for the whole-genome comparison and *r* = 0.005 (0.99) for the single-gene comparison. Only, in the nematodes we see a marginally significant correlation. However, the results for the whole-genome and for the single-gene comparison are contradictory: we observe *r* = −0.51 (0.05) for the whole-genome comparison, and *r* = 0.49 (0.06) for the single gene comparison (Figure 1B). In fact, we measure a negative correlation (*r* = −0.47 (0.08)) even between the single-gene and whole-genome substitution rates. This discrepancy may be due to the difficulties in correctly estimating substitution rates based on whole-genome comparisons, in particular with unfinished genomes, or it might be that the 18S rRNAs in the analysed nematodes are evolving unusually fast. Another striking observation in *Caenorhabditis* is the high dissimilarity in the inflated gene families. It thus appears that inflation and deflation of gene families are highly specific to species-lineages. While this holds in particular for *Caenorhabditis* (average divergence/substitution rate ratio of 24.75), it is also true—yet to a somewhat lesser extent—for the primate species (ratio 17.27). In contrast, *Drosophila* species show only a slightly inflated gene family size divergence compared to the nucleotide substitution rate (ratio 1.95). This is more than a twelve-fold difference compared to the nematodes, which cannot exclusively be attributed to different depths of the phylogenies. Genome-wide nucleotide substitution rates and estimated divergence times in generations between the most distant species differ by a factor of less than two between the nematode (av. substitution rate in all pairwise comparisons including the root of the phylogeny: 0.127) and *Drosophila* (av. substitution rate = 0.072) clades. When considering only the 18S SSU instead of the whole genome substitution rate, the numbers are even closer: 0.023 for the nematodes, and 0.019 for *Drosophila*. Hence, gene family divergence in *Drosophila* appears to be much more constrained than in nematodes. One potential reason for this observation could be linked to different reproductive modes — androdioecious hermaphrodites and gonochoristic nematodes compared to obligatory gonochoristic flies. However, whether this is indeed the case has still to be explored. Possibly, also life-style and host-association could play a role. Compared to the nematodes and flies, the primate phylogeny is much shallower in terms of generation time divergence (0.031 for the whole genome comparisons, and 0.009 for the 18S SSU comparisons). In spite of the short phylogenetic branches, gene families are considerably diverged in size. Furthermore, gene family sizes are completely uncorrelated from the substitution rate patterns, even on the relatively short time-scale of the primate phylogeny. This corroborates the view that gene family evolution is lineage- and even species-specific, and that phylogenetic signals are quickly blurred. If gene family size is shaped by selection, then the selective forces at work must be quite distinct from genome-wide background selection.

To further explore our data, we checked if gene families belonging to the different size fractions might also be functionally distinct. We find that in all classes genes in the inflated families, and the encoded proteins, have the potential to mediate interactions between cells and the environment, including host pathogen interactions (Supplementary Excel file 1).

Clearly, it is technically difficult to identify, distinguish, and to correctly assemble and map all paralogues in large gene clusters. However, even if genome assemblies and gene maps are still preliminary for many organisms, we do find a discrete number of examples—albeit surprisingly few—which are comparable in number and genomic clustering to the zebrafish NLR case.

**Figure 2:**
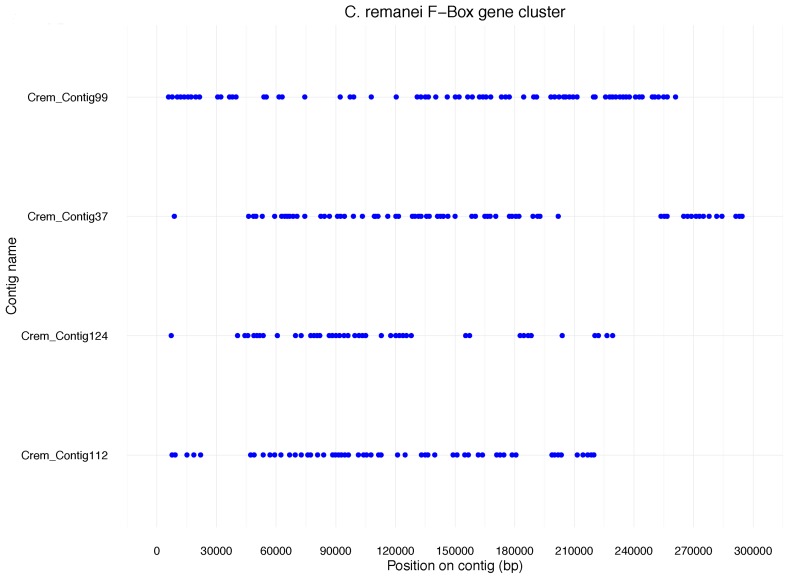
Clusters of F-Box genes found in *C. remanei*. Each dot indicates the position of an F-box gene on one of the four contigs shown in four lines.

Like NLR genes in the fish, F-box genes in nematodes are thought to play a role in pathogen resistance. We found that 688 F-box genes in *C. remanei* are located in 42 clusters containing 5 or more genes. The four largest clusters comprised over 40 genes each (*n* = 71, *n* = 69, *n* = 56, *n* = 43) (Figure 2). These data are derived from protein annotation and thus indicate functionality of the genes in *C. remanei.* The dense clustering of these genes is similar to what has previously been described for F-Box genes in *C. elegans.* However, *C. elegans* has fewer F-box genes (~260) than *C. remanei*, and, in contrast to the cases mentioned before, many of them are pseudogenes [12]. Consequently, the vast majority of F-box genes in *C. remanei* cannot be uniquely related to an *C. elegans* orthologue. This again underlines species specificity of this particular gene family expansion.

We also analysed whether NLR genes in species with known inflations could be similarly clustered as in the zebrafish. To this end, we re-compiled the list of the NLR genes in the respective species by intersecting the set of proteins we found to have a NACHT domain with those we found to have leucine rich repeats. Mapping the corresponding gene start positions on genomic scaffolds, we could identify a small cluster of NLR genes in *A. queenslandica* (5 genes) and a bigger group of 12 tightly packed NLRs in *B. floridae*. Overall, it appears that either there is no general clustering of these genes in species where they are expanded, or that the currently available genomes do not yet allow us to finally answer this question.

## 3. Conclusions

From our survey we conclude that massive expansions of gene families are rare and species-specific. For instance, regarding the NLR family there is only a very limited degree of orthology and synteny between the species *Danio rerio* and *Cyprinus carpio*. Such a situation appears to be common, given the previous analysis on shared gene families in closely related *Drosophila* species [7]. Clearly, a mechanism such as frequent tandem duplication, which promotes expansions on a short time scale, must be operating. It is, however, not clear whether duplications and losses (via pseudo-geneisation and deletion) reach a balance or whether the duplication rate is modulated depending on gene family size. An intriguing question is if functional genes can continue to proliferate unlimited when beyond a certain numerical threshold—a phenomenon, which we like to call *runaway evolution*.

Furthermore, it is currently not clear whether expansions are driven by positive selection or whether they are mostly subject to neutral genetic drift. Functional analysis of the cases, which we have compiled shows that interaction with the environment, for instance through signaling processes or immune response, is a recurring theme. This is in line with previous observations in *C. elegans* [12]. A comparison of 12 *Drosophila* genomes led to the conclusion that rapidly expanding gene families contain members which might be directly involved in speciation, e.g., functionally associated to sperm displacement or inseminating [7]. Taken together, the described functional categories hint at a potential role of positive selection, sexual selection or environmental adaptation in the initial proliferation phase of such gene families. It is then surprising that there are only so few instances of very large families. In any case, they tend to be specific to a species and show only little or no phylogenetic concordance, even between closely related species living in comparable environments. It is possible that only very few largely inflated families can be contained per genome to retain genomic stability. Additionally, these might have to remain functionally similar, due to the potential cost and to the adaptive valleys to be transgressed, when building complex regulation networks de-novo. The idea that physically close homologues might act redundantly, or in the same physiological process, is old [18,19]. Consequently, expansions may be neutral or nearly neutral for the hosting organism at least up to a certain threshold. In fact, it is hard to imagine how coordinated, and non-disruptive, regulation of the genes in very large families should evolve, if they were either highly constrained or driven by positive selection. With increasing family size, the additional costs of regulation and expression may lower a potential fitness advantage of further copies and thus keep cluster sizes under check. However, each new copy also adds to the overall duplication rate. Once a rate-threshold is surpassed, the duplication-selection-drift balance may become disrupted, with the consequence of massive gene family expansions under a possible regime of *runaway evolution*.

Returning to our example of the NLR-B30.2 genes in the zebrafish, these could act in a massive swarm in organismal defense. While still retaining their function for the organisms such genes might evolve into “genomic parasites” when continuing to proliferate, inflating and remodeling the host’s genome. Ultimately, such selfish immune-genes could be lethal for their host organism.

## 4. Methods

We calculated assembly statistics for each genome of the analysed species (Table 1) with the quast-3.1 tool to evaluate possible biases due to assembly fragmentation. However, there appears to be no correlation between assembly fragmentation and predicted number of genes in expanded families. To screen for potentially inflated gene-families we subjected proteomes to a screen with the InterProScan pipeline (v.5.7-48.0) on a local computer cluster annotating with Pfam, and PANTHER domains as well as specifically with SUPERFAMILY, and GENE3D, as these two pipelines have been reported to detect NLR genes based on structural models [15].

### 4.1. Identification of NLR Genes

Initially, we scanned the InterProScan output from the sponge, the carp, the sea urchin, and the lancet for NACHT domains and leucine rich repeats using the respective Pfam domains. We only designated proteins with both annotations as NLR genes. We found that in *Strongylocentrotus* and *Amphimedon*, many LRRs could only be retrieved by the additional use of Gene3D, and Superfamily annotations, which apply structure based HMMs. We supplemented our search with PANTHER IDs. Although PANTHER often found many additional NLRs, most of the proteins identified by it were lacking NACHT and LRR domains and were therefore discarded.

### 4.2. Indentification of Species Specific Gene Expansions

To find new, potentially clustered gene families, we implemented a Fisher’s exact test with Benjamini and Hochberg correction in R and Python (Table 3). In this way, we tested for enrichment of Pfam domains in pairwise comparisons between sets of species in Caenorhabditis and Drosophila as well as between the great apes (Table 2). We kept domains with odds ratio ≥5 or ≤0.3 and a domain count greater than 20 in either of the compared species. For each of the collected Pfam IDs, we counted the proteins for all primates, nematodes and flies. Domains present in more than 200 proteins were mapped to GO terms from the InterProScan analysis. We then kept only those candiates where the retrieved annotation likely represents families of proteins and not only domains found in many different gene families.

We used the alignment free measure Kr [20] and the more recent Andi method [17] to calculate nucleotide substition rates based on 18S ribosomal sequences for each of the three taxa, Caenorhabditis, Drosophila, “great apes”. The same approach was also applied to genome wide comparisons. We used neighbour-joining [21] to infer trees from distance matrices. Gene family divergence was calculated as standardised squared difference in gene numbers, summed across families.

### 4.3. Detecting Gene Clusters

We checked if the most inflated gene families in our analysis (NLR, F-box, Fibronectin) are clustered in the genomes by screening for start positions on scaffolds based on the downloaded GFF files. We considered a group of more than 4 genes with a distance of less than 10 kb to be clustered.

For all *C. remanei* F-Box clusters we extracted the genomic regions (ranging from the start coordinates of the first gene in the cluster to the end position of the last gene) and aligned these with the *C. elegans* genome using MUGSY (v1r2.2) [22]. As an extra measure to determine genomic homology we extracted all F-box genes independently and blasted them against the *C. elegans* genome using NCBI BLAST+ v2.2.31.

## Figures and Tables

**Table 1:** List of all databases which were mined for genome, proteome and annotation files. Corresponding genome assembly statistics.

Species	Database	N50	Contigs	Largest Contig	Total Length
**Sponges**					
*Acropora digitifera*	NCBI (adi_v0.9)	41904	18834	0.48 Mb	412 Mb
*Amphimedon queenslandica*	Ensembl (Aqu1.29)	120365	13397	1.9 Mb	166.7 Mb
**Fishes**					
*Cyprinus carpio*	Carpbase (v2.0)	7828866	9376	29.1 Mb	1713.7 Mb
*Danio rerio*	Ensembl (Zv9)	54093808	1133	77.3 Mb	1412.5 Mb
**Lancelets**					
*Branchiostoma floridae*	NCBI (v2.0)	2586727	398	11.5 Mb	521.9 Mb
**Echinoderms**					
*Strongylocentrotus purpuratus*	NCBI (Spur_4.2)	421711	27578	2.5 Mb	989.4 Mb
**Flies**					
*Drosophila erecta*	Flybase (r1.04)	18748788	5103	26.6 Mb	152.7 Mb
*Drosophila melanogaster*	Flybase (r6.07)	25286936	1870	32.1 Mb	143.7 Mb
*Drosophila pseudoobscura*	Flybase (r3.03)	12541198	4463	30.8 Mb	152.6 Mb
*Drosophila simulans*	Flybase (r2.01)	23539531	2601	27.2 Mb	123.6 Mb
**Roundworms**					
*Caenorhabditis angaria*	Wormbase (WS249)	87708	11453	0.87 Mb	99.01 Mb
*Caenorhabditis brenneri*	Wormbase (WS249)	381961	3305	4.1 Mb	190.4 Mb
*Caenorhabditis briggsae*	Wormbase (WS249)	17485439	12	21.5 Mb	108.4 Mb
*Caenorhabditis elegans*	Wormbase (WS249)	17493829	7	20.9 Mb	100.3 Mb
*Caenorhabditis japonica*	Wormbase (WS249)	94149	18808	1.1 Mb	166.3 Mb
*Caenorhabditis remanei*	Wormbase (WS249)	435512	3670	4.5 Mb	145.4 Mb
**Primates**					
*Gorilla gorilla*	NCBI (v3.1)	145327772	49216	229.5 Mb	3035 Mb
*Pongo abelii*	NCBI (v2.0.2)	135191526	61534	229.9 Mb	3411 Mb
*Pan troglodytes*	NCBI (v2.1.4)	143986469	24128	247.5 Mb	3309 Mb
*Pan paniscus*	NCBI (v1.1)	144709823	10209	247.9 Mb	3286 Mb
*Homo sapiens*	NCBI (GRCh38.p5)	145138636	517	145.1 Mb	3230 Mb

**Table 2:** NACHT-domain and leucine-rich-repeat-containing (NLR) gene candidates (encoding for NACHT domains and LRRs) identified with Pfam, Gene3D and Superfamily in interproscan. Supplemented through PANTHER annotations.

Species	NLR Genes
*Acropora digitifera*	276
*Amphimedon queenslandica*	95
*Branchiostoma floridae*	44
*Cyprinus carpio*	153
*Strongylocentrotus purpuratus*	65

**Table 3:** Pairwise comparisons in Fisher’s exact text to identify enriched Pfam domains.

Species 1	Compared with
*Homo sapiens*	*Gorilla gorilla, Pongo abelii, Pan troglodytes, Pan paniscus*
*Pan troglodytes*	*Pongo abelii*
*Gorilla gorilla*	*Pan paniscus*
*Drosophila melanogaster*	*Drosophila erecta, Drosophila pseudoobscura, Drosophila simulans*
*Drosophila simulans*	*Drosophila pseudoobscura, Drosophila erecta*
*Caenorhabditis elegans*	*Caenorhabditis angaria, Caenorhabditis brenneri, Caenorhabditis briggsae, Caenorhabditis japonica, Caenorhabditis remanei*
*Caenorhabditis remanei*	*Caenorhabditis angaria*
*Caenorhabditis japonica*	*Caenorhabditis briggsae, Caenorhabditis brenneri*

## Supplementary Materials

Supplementary File 1

## Abbreviations

The following abbreviations are used in this manuscript:

NLR: NACHT-domain and leucine-rich-repeat-containing
